# A new electrochemical angular microaccelerometer with integrated sensitive electrodes perpendicular to flow channels

**DOI:** 10.1038/s41378-022-00411-0

**Published:** 2022-07-12

**Authors:** Bowen Liu, Tian Liang, Wenjie Qi, Anxiang Zhong, Mingwei Chen, Yulan Lu, Jian Chen, Deyong Chen, Junbo Wang

**Affiliations:** 1 The State Key Laboratory of Transducer Technology, Aerospace Information Research Institute, Chinese Academy of Sciences, 100190 Beijing, China; 2 The School of Electronic, Electrical and Communication Engineering, University of Chinese Academy of Sciences, 100049 Beijing, China

**Keywords:** Electrical and electronic engineering, Sensors

## Abstract

A new electrochemical angular microaccelerometer with integrated sensitive electrodes perpendicular to flow channels was developed in this paper. Based on a liquid inertial mass, an incoming angular acceleration was translated into varied concentrations of reactive ions around sensitive microelectrodes, generating a detection current. Key structural parameters of the sensitive microelectrodes were designed and compared based on theoretical analysis and numerical simulations. An angular microaccelerometer incorporating sensitive microelectrodes was then fabricated, assembled and characterized, producing a sensitivity of 338 V/(rad/s^2^), a −3 dB bandwidth of 0.01–10 Hz and a noise level of 4.67 × 10^−8^ (rad/s^2^)/Hz^1/2^ @ 1 Hz. These performances were better than their commercial counterparts based on traditional electrodes and previously reported microaccelerometers based on microsensitive electrodes in parallel with flow channels, which can be applied to measure rotational accelerations in earthquakes and buildings.

## Introduction

Rotational components exist widely in earthquakes^1,2^, and a large number of studies and records have shown that rotational components have destructive effects on building safety^3,4^. Angular acceleration is the key parameter used to describe rotation components, and the accurate measurement of angular acceleration is of great significance for the study of rotational components in seismology and the anti-seismic design of buildings^5,6^.

At present, there are mainly two types of angular accelerometers, which are based on solid and liquid inertial masses^7^. Among them, the basic principle of angular accelerometers leveraging solid inertial masses is mostly based on energy exchanges of capacitances or moving coils^8–11^. However, due to problems such as limited bandwidth, poor impact resistance, high noise level and complex fabrication, this kind of angular accelerometer cannot meet the requirements of the above seismology field^1,12^.

An angular accelerometer based on a liquid inertial mass mainly includes two energy conversion methods based on an interface effect and an electrochemical reaction. An angular accelerometer based on the interface effect of the electrical double layer cannot be applied in earthquake monitoring applications because of its low sensitivity and limited bandwidth^13,14^. Meanwhile, an angular accelerometer based on the electrochemical reaction is suitable for the detection of angular accelerations in seismology because of its large bandwidth, high sensitivity and lack of mechanical noise.

Studies on electrochemical angular accelerometers have mainly included two types^6,15–19^ based on traditional^18^ and microfabricated electrodes^19^. Angular accelerometers based on traditional electrodes are difficult to manufacture, and electrode parameters cannot be adjusted, leading to compromised performances^20,21^. For angular accelerometers based on sensitive microelectrodes, since they are parallel to flow channels, they suffer from limited electrode areas and slow electrolyte velocity, leading to compromised sensitivities.

To solve the above problems, this study developed a new electrochemical angular microaccelerometer with integrated sensitive microelectrodes perpendicular to flow channels, which can greatly improve the key performance of the sensor by optimizing the structure and placement of the sensitive electrodes. The following sections of this article include materials and methods, results and a discussion, and a conclusion.

## Materials and methods

### Structure and working principle

**Fig. 1:**
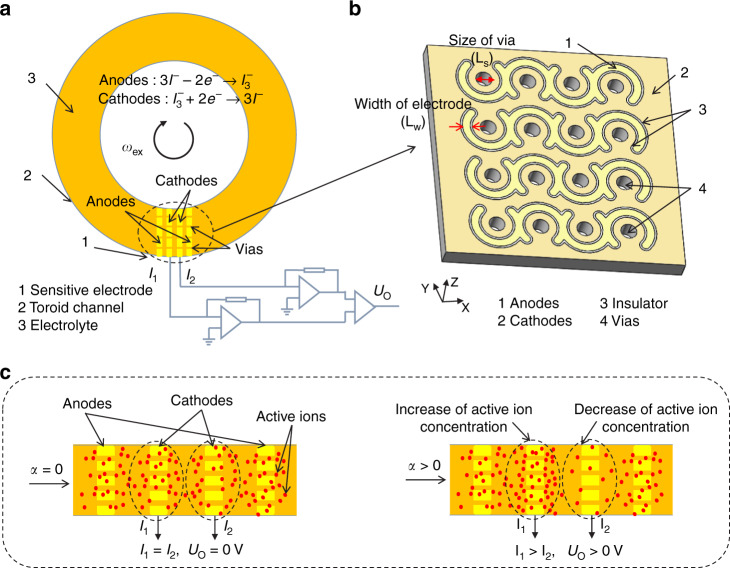
Main structure and working principle of the new electrochemical angular microaccelerometer with integrated sensitive microelectrodes perpendicular to flow channels. **a** Schematic of the developed electrochemical angular microaccelerometer and the sensitive electrodes represented the four-electrode structure. **b** A 3D setup of the four-electrode structure, where a pair of anodes and cathodes were distributed on the front and back sides and run through by thousands of vias filled with an electrolyte solution. **c** The working principle of the MEMS electrochemical angular accelerometer in which in response to an external angular vibration, reactive ions in the electrolyte flow along the toroid channel and the vias on the sensitive micro electrodes.

The main structure and working principle of the new electrochemical angular microaccelerometer with integrated sensitive microelectrodes perpendicular to flow channels are shown in Fig. 1a. The sensitive microelectrodes were made of four-electrode structures, which were composed of two pairs of cathodes and anodes arranged in an anode-cathode-cathode-anode order. In addition, the toroid channel was filled with an electrolyte solution (e.g., potassium iodide and iodine) as a liquid inertial mass sensitive to external angular acceleration.

The structures of the sensitive electrodes and their placements in relation to the flow channels have an important influence on the sensor performance. Therefore, integrated sensitive microelectrodes perpendicular to flow channels were presented in this study. As shown in Fig. 1b, the sensitive electrodes were implemented on a silicon substrate where the entire electrode was in a mesh structure. A pair of cathodes and anodes were distributed on the front and back sides of the silicon substrate and were separated by an insulating ring of SiO_2_. In addition, they were connected to the surrounding electronic circuit by leads on the left and right sides. Furthermore, the vias were distributed on the cathode so that the reactive ions in the electrolyte solution were along the direction of the anode-cathode-cathode-anode setup. Note that vias played a certain role in the compression channels, which further increased the flow velocity of electrolytes near the electrodes. More specifically, on each side of the silicon substrate, the cathodes and anodes around each via were directly distributed, which was used to effectively improve the utilization of reactive ions to increase the electrode sensitivity, and thus key electrode and via parameters could be adjusted.

More specifically, the working principle of the MEMS electrochemical angular accelerometer is shown in Fig. 1c, where a voltage of 0.3 V was applied between each pair of anodes and cathodes, and then the oxidation-reduction reactions that occurred on the anodes and cathodes were $3I^ - - 2e^ - \to I_3^ -$ and $I_3^ - + 2e^ - \to 3I^ -$ to generate an output current^21,22^. When there was no angular acceleration outside, the reactive ions (red dots, I_3_^−^) formed a stable concentration gradient distribution around the cathodes. Therefore, the output current of the two cathodes was the same, and the differential voltage U_O_ was zero. When the external angular acceleration was caused by a seismic vibration, the electrolyte moved relative to the sensitive microelectrodes through the toroid channel and the vias on the sensitive electrodes, causing the concentration of reactive ions near the two cathodes to change differentially, leading to a differential current output, and the output current signal of the fabricated sensor was converted into a voltage signal through an external transimpedance.

### Numerical simulation

#### Modeling

The detection process of the electrochemical angular accelerometer was composed of two energy conversion modules: a mechanical module and an electrochemical module. The mechanical module was a vibration system composed of an electrolyte solution (liquid inertial mass) and a toroid channel, which converted the external signals of angular vibrations into variations of electrolyte velocities near the electrodes. The electrochemical module was composed of the sensitive microelectrodes and the nearby reactive ions, which converted the velocities of electrolytes into output currents through an electrochemical reaction. In this study, mechanical and electrochemical modules were established through finite element simulation, and the key parameters of microelectrodes (size of via and width of electrode) were studied.

**Fig. 2:**
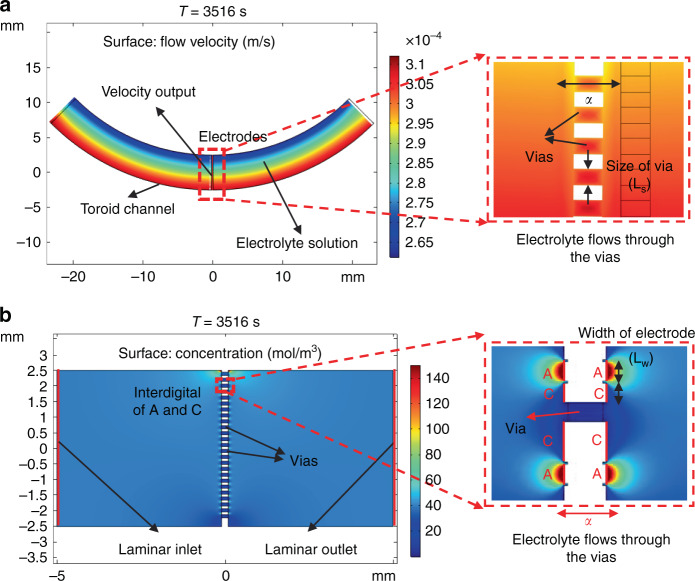
Numerical simulations of the electrochemical angular microaccelerometer with integrated sensitive microelectrodes perpendicular to flow channels. **a** Numerical simulations of the mechanical module converting the signal of external angular vibration into the vibration of the electrolyte solution. **b** Numerical simulations of the electrochemical module converting the electrolyte vibration into electrochemical reactions on the sensitive microelectrodes for electrical outputs.

The two-dimensional simulation models of the mechanical and electrochemical modules established in this study are shown in Fig. 2a, b. First, in the simulation of the mechanical module, the dynamic viscosity of the electrolyte was set to 8.28 × 10^−4^ Pa·s, and the width of the flow channels was set to 5 mm. The physical field of “rotating machine and laminar flow” was used to input the signal of sinusoidal angular vibration in the rotating domain, and the frequency was swept from 0.01–10 Hz. In addition, the boundary conditions were open boundaries for both sides of the model, a normal stress of 0 N/m^2^, and no slippery channel walls. Then, the model was solved by a transient solver with full coupling, and the output parameters of these simulations were the electrolyte velocities near the microelectrodes.

In the simulation of the electrochemical module, the width of flow channels, the space of sensitive electrodes and the dynamic viscosity of electrolyte were defined as 5 mm, 10 μm, and 8.28 × 10^−4^ Pa·s, respectively. The coupled physical field of “laminar flow” and “tertiary current analysis” was used for simulation, and the signal of sinusoidal angular vibration was used as the input at the inlet of laminar flow, with the frequency swept from 0.01–10 Hz. The boundary conditions were an anode voltage of 0.3 V, a cathode voltage of 0 V, no viscous stress at the channel outlet, and constant concentrations at the inlet and outlet. Then, the model was solved by a transient solver with full coupling, and the output parameters of these simulations were the differential current outputs of the cathodes.

## Results

In this study, the key parameters of the integrated sensitive microelectrodes were simulated, including the size of the via (L_s_ of 150, 100 and 80 μm) and the width of the electrode (L_w_ of 20 and 100 μm). The size of the via could affect the hydrodynamic resistance (R_h_) of the device, which mainly affected the mechanical module, while the width of the electrode could affect the electrochemical reaction area and mainly affected the electrochemical module.

**Fig. 3:**
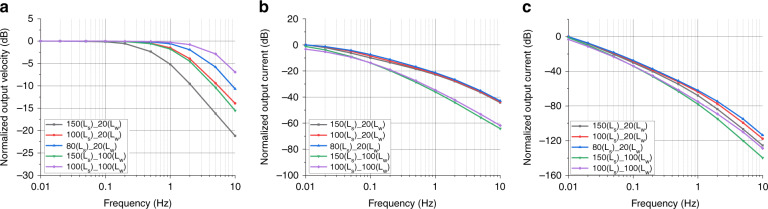
Simulation results of the electrochemical angular microaccelerometer with integrated sensitive microelectrodes perpendicular to flow channels. **a** Simulation results of the mechanical module, where the *x*-axis represents the input frequency of angular vibration and the y-axis represents the output velocity of electrolyte near the electrodes. **b** Simulation results of the electrochemical module, where the x-axis represents the velocity frequency of the electrolyte solution and the *y*-axis represents the output current of electrodes. **c** Overall simulation results of the mechanical and the electrochemical modules, where the *x*-axis represents the frequency of the angular vibration and the y-axis represents the output current of electrodes.

Figure 3a shows the simulation results of the mechanical module, where the *x*-axis represents the frequency of the external angular vibration and the *y*-axis represents the velocity of the electrolyte near the electrodes, where five curves correspond to sensitive microelectrodes with different electrode parameters. The simulation results showed that the mechanical module can be considered a low-pass link, which was consistent with the theoretical analysis of the electrochemical angular accelerometer^6^. Under the width of the electrode (L_w_) at 20 μm or 100 μm, when the size of via (L_s_) was reduced from 150 μm to 100 μm and 80 μm, the high-frequency turning points of the curves were observed to shift to the right, and the same conclusion could be obtained when the width of electrode (L_w_) was fixed to 100 μm and the size of via (L_s_) was reduced from 150 μm to 100 μm. This is because reducing the size of via (L_s_) could increase the hydrodynamic resistance of the device, thereby delaying the decline in high frequencies, which was consistent with theoretical analysis of the mechanical module^6,23^. In addition, the overall analysis of the five curves was also in line with the influence of the hydrodynamic resistance on the high-frequency turning point, where the hydrodynamic resistances of the five curves from top to bottom were estimated as 3.75 × 10^11^ Pa∙s/m^3^, 3.05 × 10^11^ Pa∙s/m^3^, 1.35 × 10^11^ Pa∙s/m^3^, 7.40 × 10^10^ Pa∙s/m^3^, and 3.33 × 10^10^ Pa∙s/m^3^, respectively.

Figure 3b shows the simulation results of the electrochemical module, where the *x*-axis represents the frequency of electrolyte velocity and the *y*-axis represents the current of the electrodes. From the simulation results, it was observed that the increasing frequency of the electrolyte velocity reduced the output current of the electrodes, which was consistent with theoretical analysis^6^. In addition, when other parameters were the same, reducing the width of the electrode (L_w_) from 100 μm to 20 μm increased the output current of the electrodes (normalizing the electrode area), which was caused by the increase in the electrode area participating in effective electrochemical reactions under the same substrate area, which was consistent with previous studies^19^.

Figure 3c shows the overall simulation results of the mechanical and electrochemical modules, where the *x*-axis represents the vibration frequency of external angular acceleration and the *y*-axis represents the output current of the electrodes. The simulation results showed that on the one hand, the turning point of the high-frequency decline can be changed through the size of via (L_s_), and on the other hand, the sensitivity of the device can be adjusted by modifying the width of electrode (L_w_).

### Fabrication

**Fig. 4:**
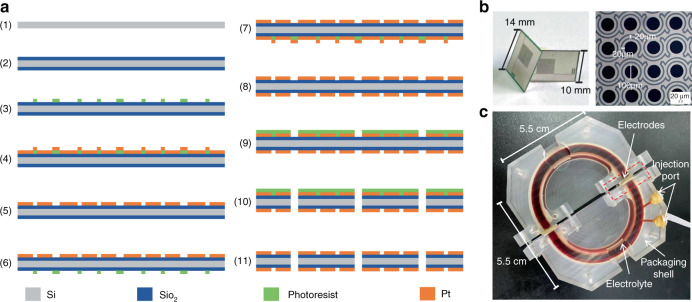
Fabrication of the integrated sensitive microelectrodes as the sensing component of the electrochemical angular microaccelerometer. **a** Schematic of fabrication process of the integrated sensitive microelectrodes. Prototyped images of (**b**) fabricated sensitive microelectrodes with detailed electrode structures highlighted and **c** an assembled electrochemical angular microaccelerometer with mechanical compression and electrolyte injection.

The fabrication of the integrated sensitive microelectrodes developed in this study adopted a conventional MEMS process, where the four-electrode structure was fabricated on one silicon wafer (see Fig. 4a). Key steps included (1) silicon substrate cleaning, (2) oxidation, (3) lithography, (4) evaporation of platinum, (5) lift-off to form electrode pairs on the front, (6) aligned lithography on the back, (7) evaporation of platinum on the back, (8) lift-off to form electrode pairs on the back, (9) aligned lithography on the front, (10) RIE/DRIE, and (11) removal of photoresist to obtain the integrated sensitive electrodes. Figure 4b shows a prototype of the integrated sensitive microelectrodes, highlighting microelectrodes. After positioning the sensitive microelectrodes perpendicular to the flow channels through mechanical compression after electrolyte injection, a new electrochemical angular microaccelerometer with integrated sensitive electrodes perpendicular to the flow channels was obtained, as shown in Fig. 4c.

## Results and discussion

### Sensitivity and bandwidth

The characterization of the developed electrochemical angular accelerometers was realized on a standard angular acceleration turntable, which was used to provide angular acceleration signals with frequencies of 0.01–10 Hz and amplitudes of 0.003–7.896°/s^2^. To validate the simulation results of the electrode parameters, devices with five different electrode parameters were tested in this study.

**Fig. 5:**
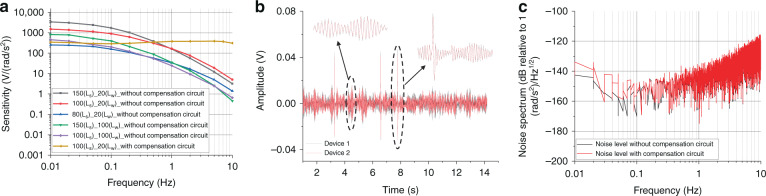
Characterization results of the developed electrochemical angular microaccelerometer with integrated sensitive electrodes perpendicular to flow channels. **a** Results of sensitivity characterization of the developed devices, in which the *x*-axis represents the frequency of angular acceleration and the y-axis represents the sensitivity. **b** Results of correlation characterization of two devices incorporating sensitive microelectrodes with size of via (Ls) at 100 μm and width of electrode (Lw) at 20 μm. **c** Results of noise characterization of the device without and with a compensation circuit.

Figure 5a shows the characterization results of the developed electrochemical angular microaccelerometer with integrated sensitive electrodes perpendicular to flow channels, in which the *x*-axis represents the frequency of angular acceleration and the *y*-axis represents the sensitivity of the sensor, which was defined as the output voltage of the fabricated angular accelerometer divided by the input amplitude of the angular acceleration. It was observed that under the width of electrode (L_w_) at 20 μm, reducing the size of via (L_s_) from 150 μm to 100 μm and 80 μm with the increase of the hydrodynamic resistance could move the turning point of high-frequency decline to the right, which was consistent with the simulation results. Note that it was also found that the sensitivity level decreased to a certain extent with the reduction of the size of via (L_s_), and the overall sensitivity curve of the device moved downward, which was caused by slowing down the electrolyte flow in the actual test.

Similarly, when the width of electrode (L_w_) was fixed at 100 μm and the size of via (L_s_) was reduced from 150 μm to 100 μm, the same results were found. In addition, when the size of via (L_s_) was fixed at 150 μm or 100 μm, reducing the width of electrode (L_w_) from 100 μm to 20 μm increased the sensitivity of the electrodes, which was consistent with the simulation results. Finally, when the position of the high-frequency turning point and sensitivities were taken into consideration, the size of via (L_s_) at 100 μm and the width of electrode (L_w_) at 20 μm were selected as the optimal electrode parameters in the electrochemical angular accelerometers.

In addition, the yellow line in Fig. 5a shows the test results of the sensitive microelectrodes in which the size of via (L_s_) was 100 μm and the width of electrode (L_w_) was 20 μm with a compensation circuit^24^. After compensation by a third-order circuit, the device can achieve a sensitivity of 338 V/(rad/s^2^) and a bandwidth of 100 s–10 Hz. By comparing with the commercially available counterpart based on traditional electrodes^18^ and the counterparts based on sensitive microelectrodes parallel to flow channels^19^ (see Table 1), the devices fabricated in this study demonstrated a higher sensitivity (338 vs. 8 vs. 22 V/(rad/s^2^)) and a wider bandwidth (100 s–10 Hz vs. 50 s–10 Hz vs. 50 s–10 Hz), indicating that the angular accelerometer developed in this study demonstrated significantly better performances in the low-frequency domain.

**Table 1:** Comparison of key parameters of the angular accelerometer

Characteristic	Unit	Counterpart in ref. ^18^	Counterpart in ref. ^19^	This study
Sensitivity	V/(rad/s^2^)	8	22	338
Bandwidth (−3 dB)	–	50 s–10 Hz	50 s–10 Hz	100 s–10 Hz
Noise level	(rad/s^2^)/Hz^1/2^	5.62 × 10^−6^ @ 1 Hz	8.91 × 10^−7^ @ 1 Hz	4.67 × 10^−8^ @ 1 Hz

### Correlation and noise level

In terms of correlation characterization, two devices incorporating sensitive microelectrodes with a size of via (L_s_) at 100 μm and a width of electrode (L_w_) at 20 μm were positioned on the same ground, where the output signals of the devices were collected by the data acquisition card. The characterization results are shown in Fig. 5b, where the *x*-axis represents the time and the *y*-axis represents the voltage amplitude of the device. The two devices demonstrated high correlations, which were quantified as 0.99.

In terms of noise-level characterization, when the device with the size of via (L_s_) at 100 μm and the width of electrode (L_w_) at 20 μm was positioned in a low-noise room, it was sampled by a data acquisition card at night. Figure 5c shows the characterization results of the noise level with and without a compensation circuit, where the *x*-axis represents the vibration frequency of the angular vibration and the *y*-axis represents the power spectral density in the unit of applied angular acceleration. More specifically, the noise levels of the developed device without and with the compensation circuit were quantified as 4.62 × 10^−8^ (rad/s^2^)/Hz^1/2^ (−146.70 dB) vs. 7.12 × 10^−8^ (rad/s^2^)/Hz^1/2^ (−142.94 dB) @ 0.01 Hz, 4.32 × 10^−8^ (rad/s^2^)/Hz^1/2^ (−147.28 dB) vs. 4.67 × 10^−8^ (rad/s^2^)/Hz^1/2^ (−146.61 dB) @ 1 Hz, 3.52 × 10^−7^ (rad/s^2^)/Hz^1/2^ (−129.08 dB) vs. 5.28 × 10^−7^ (rad/s^2^)/Hz^1/2^ (−125.54 dB) @ 10 Hz, in which the noise level with a compensation circuit was slightly higher than that without the compensation circuit. In addition, compared with the currently available commercial counterpart based on traditional electrodes^18^ and a counterpart based on sensitive microelectrodes parallel to flow channels^19^ (see Table 1), the device with a compensation circuit manufactured in this study had a lower noise level (4.67 × 10^−8^ vs. 5.62 × 10^−6^ vs. 8.91 × 10^−7^ @ 1 Hz).

## Conclusion

In this study, numerical simulation, fabrication and characterization of an electrochemical angular microaccelerometer with integrated sensitive electrodes perpendicular to flow channels were conducted. Based on the results of experimental characterization, the angular microaccelerometer produced a sensitivity of 338 V/(rad/s^2^), a −3 dB bandwidth of 100 s–10 Hz and a noise level of 4.67 × 10^−8^ (rad/s^2^)/Hz^1/2^ @ 1 Hz. These performances were better than those of commercial counterparts based on traditional electrodes and previously reported microaccelerometers based on microsensitive electrodes in parallel with flow channels, which could provide a new perspective for the monitoring of angular vibrations in seismology.
